# Dual CDK and MEK Inhibition potentiates CD8^+^ T cell-mediated antitumor immunity by inducing pyroptotic cell death in high-mutational head and neck cancer

**DOI:** 10.1186/s13046-025-03557-7

**Published:** 2025-11-06

**Authors:** Fanghui Chen, Fang Yang, David O. Popoola, Jianqiang Yang, Chris Tang, Alexis Payne, Lynn Zhang, Nicole C. Schmitt, Jin Xie, Nabil F. Saba, Yamin Li, Yong Teng

**Affiliations:** 1 Department of Hematology and Medical Oncology, Emory University, 201 Dowman Dr, Atlanta, GA 30322 USA; 2 Department of Pharmacology, State University of New York, Upstate Medical University, Syracuse, NY 13210 USA; 3 Department of Otolaryngology, Emory University, Atlanta, GA 30322 USA; 4 Winship Cancer Institute of Emory University, Atlanta, GA 30322 USA; 5 Department of Chemistry, University of Georgia, Athens, GA 30602 USA; 6 Wallace H. Coulter Department of Biomedical Engineering, Georgia Institute of Technology and Emory University, Atlanta, GA 30322 USA

**Keywords:** HNSCC, AZD5438, PD0325901, Lipid nanoparticles, Pyroptosis, Combination therapy, High mutational burden

## Abstract

**Background:**

HPV-negative (−) head and neck squamous cell carcinoma (HNSCC) is a highly heterogeneous cancer characterized by high mutational burden, an immunosuppressive microenvironment, and poor response to standard therapies. These features highlight the urgent need for novel and more effective treatment strategies.

**Methods:**

Drug sensitivity prediction was performed using integrated datasets from TCGA, GDSC, and CCLE. To assess the therapeutic potential and underlying mechanisms of combining the CDK inhibitor AZD5438 with the MEK1/2 inhibitor PD0325901, we employed a comprehensive panel of HNSCC models, including established cell lines, orthotopic mouse tumor models, and patient-derived organoids (PDOs). Lipid nanoparticles (LNPs) were engineered to co-deliver both agents into the same cancer cell populations. The tumor secretome was profiled using biotinylation coupled with liquid chromatography-mass spectrometry (LC-MS). Molecular alterations were examined by immunofluorescence, immunohistochemistry, ELISA, flow cytometry, and Western blot.

**Results:**

Our bioinformatics analysis identified AZD5438 and PD0325901 as two of thirteen candidate drugs whose sensitivity is consistently associated with the five most frequently mutated genes in HPV (−) HNSCC. Notably, among these candidates, AZD5438 and PD0325901 exhibited the lowest correlation in their sensitivity profiles, suggesting complementary mechanisms of action. In experimental models, the combination of AZD5438 and PD0325901 not only outperformed either monotherapy in suppressing tumor growth but also augmented CD8⁺ T cell-mediated antitumor immunity by promoting caspase-8/gasdermin E-dependent pyroptosis. Furthermore, in both orthotopic tumor-bearing mice and PDOs, the LNP-encapsulated drug combination produced significantly greater therapeutic efficacy compared with the free drug formulation.

**Conclusions:**

Our findings indicate that the combination of AZD5438 and PD0325901 holds therapeutic potential for the treatment of HPV (−) HNSCC, particularly in tumors with a high mutational burden. By targeting complementary pathways, this combination may improve treatment outcomes in this aggressive cancer subtype.

**Supplementary Information:**

The online version contains supplementary material available at 10.1186/s13046-025-03557-7.

## Background

Head and neck squamous cell carcinoma (HNSCC) represents a highly heterogeneous group of malignancies originating from the mucosal epithelium of diverse anatomical regions within the head and neck. The complexity of effectively managing HNSCC arises not only from its pronounced heterogeneity in anatomical origin but also from its elevated levels of genetic instability [1, 2]. The genetic architecture of HNSCC is notably intricate, characterized by the accumulation of numerous mutations throughout tumorigenesis and progression. These genetic alterations, particularly in key oncogenes and tumor suppressors, not only complicate mechanistic insights but also impede progress toward effective targeted treatments [3–5].

High mutational burdens in cancers like melanoma, lung cancer, and HNSCC present a dual-edged sword, complicating treatment strategies while also providing potential avenues for therapeutic innovation. In malignancies driven by recurrent oncogenic mutations (e.g., *EGFR* in lung cancer, *BRAF* in melanoma), targeted therapies have demonstrated considerable clinical efficacy [6, 7]. However, the high mutation rates in these cancers often lead to resistance mechanisms, including secondary mutations, activation of alternative signaling pathways, or tumor plasticity. To address these issues, combination therapies targeting multiple molecular pathways are under investigation to mitigate or even overcome the emergence of resistance. Although the inherent genetic instability of these cancers promotes adaptability and resistance to conventional therapies, it also exposes therapeutic vulnerabilities that can be strategically targeted.

AZD5438 is an orally bioavailable cyclin-dependent kinase (CDK) inhibitor that exhibits multi-kinase inhibitory activity, targeting cyclin E-CDK2, cyclin A-CDK2, cyclin B1-CDK1, p25-CDK5, cyclin D3-CDK6, and cyclin T-CDK9 [8, 9]. It has been demonstrated to effectively suppress the phosphorylation of key CDK substrates, including retinoblastoma protein (Rb), nucleolin, protein phosphatase 1α, and RNA polymerase II, leading to cell cycle arrest at the G1, S, and G2-M phases [10, 11]. AZD5438 has not been evaluated in HNSCC; however, another CDK inhibitor, palbociclib, has been tested in combination with cetuximab in patients with platinum-resistant or cetuximab-resistant HPV-negative (−) HNSCC, demonstrating promising clinical activity [12]. PD0325901 is a highly selective inhibitor of the mitogen-activated protein kinase (MAPK)/extracellular signal-regulated kinase (ERK) pathway, specifically targeting MEK1 and MEK2 [13, 14]. By inhibiting these kinases, PD0325901 disrupts essential signaling cascades critical for cell proliferation and survival in cancers driven by mutations in the Ras/Raf/MEK/ERK pathway. In HNSCC cells, co-treatment of PD0325901 with the PI3K/mTOR inhibitor PF-384 overcame resistance by blocking ERK signaling and enhanced anti-proliferative, pro-apoptotic, and anti-angiogenic effects in vitro and in vivo [15]. To the best of our knowledge, no studies have reported on the therapeutic combination of AZD5438 and PD0325901 in any cancer type.

HPV (−) and HPV-positive (+) HNSCC represent distinct clinical entities [16, 17]. HPV (+) HNSCC exhibit a unique genetic landscape characterized by specific mutations, whereas the mutational profile of HPV (−) HNSCC closely resembles that of lung and esophageal squamous cell carcinomas [18]. In this study, bioinformatics analysis identified AZD5438 and PD0325901 as two of 13 drugs whose sensitivity was commonly associated with the five most frequently mutated genes in HPV (−) HNSCC. Drug correlation analysis revealed that these two agents had the lowest correlation factor (CF) in their sensitivity profiles among the 13 drugs identified, suggesting potentially complementary mechanisms of action. The combination of AZD5438 and PD0325901 exhibited superior tumor-suppressive efficacy compared to either agent alone and further amplified CD8⁺ T cell-mediated antitumor immunity through the induction of pyroptosis, a form of programmed cell death characterized by its pro-inflammatory nature [19, 20]. Nanoparticle-mediated co-delivery encapsulates both drugs within the same carrier, ensuring simultaneous delivery to the same tumor cells. This approach improves pharmacokinetic compatibility, enhances drug stability, synchronizes intracellular release, and increases the likelihood of synergistic action, while reducing off-target toxicity compared to administering the drugs separately [21]. In both orthotopic tumor-bearing mice and patient-derived organoids (PDOs), co-delivery of the two agents via lipid nanoparticles (LNPs) produced markedly greater therapeutic benefits than administration of the free drug combination. These results demonstrate that the dual inhibition strategy using AZD5438 and PD0325901 offers a promising therapeutic approach for HPV (−) HNSCC, especially in tumors characterized by a high mutational burden.

## Methods

### Correlation analysis between gene mutations and expression in TCGA HNSCC cohort

Gene mutation data were extracted from TCGA HNSCC dataset using ‘TCGAbiolinks’ R package with ‘Simple Nucleotide Variation’ and ‘Masked Somatic Mutation’ based on ‘open’ data. The ‘Maftools’ package was used to count gene mutations. A gene mutation-expression matrix was constructed by integrating the gene mutation matrix and gene expression matrix based on patient ID and gene symbol. The gene mutation-expression matrix for the top 10 most frequently mutated genes was divided into two subgroups based on the gene status of wildtype or mutant.

### Drug sensitivity prediction using integrative data from TCGA and GDSCs datasets

Drug sensitivity data were obtained from the GDSCs dataset (367 compounds in GDSC1 dataset and 198 compounds in GDSC2 dataset) and the drug sensitivity matrix was constructed based on patient ID and drug name. Clinical drug sensitivity information for HNSCC patients was obtained from the NCI Genomic Data Commons portal. Drug names and sensitivity information were retrieved from the clinical metadata and edited for typographical and spelling errors and to harmonize commercial names and molecular drug names. Differences in drug sensitivity between the two subgroups (wildtype vs. mutant) were evaluated using “limma” and “oncoPredict” R package. *p* < 0.05 was considered statistically significant.

### Drug sensitivity prediction using integrative data from CCLE and GDSCs datasets

The gene expression data of 56 HNSCC cells lines were downloaded from CCLE (http://sites.broadinstitute.org/ccle) dataset. A gene expression matrix was constructed based on cell line name and gene symbol. A drug sensitivity matrix was constructed based on the cell data obtained from the GDSCs dataset (a total of 987 cell lines in GDSC1 dataset and 805 cell lines in GDSC2 dataset). The gene expression and drug sensitivity matrixes were integrated and normalized by Log10 to assess correlation between drug sensitivity and HNSCC cell lines using ‘oncoPredict’, ‘gtools’, ‘reshape2’, and ‘ggpubr’ R package [22]. No multiple comparison correction was applied when analyzing correlations between gene expression and drug sensitivity, and the significance threshold used in the oncoPredict package was defined as *p* < 0.05. In this study, if the IC_50_ was exceptionally high (>100µM), the drug was deemed to have no effect on tumor cells. Conversely, if the IC_50_ was extremely close to 0 µM, the drug was considered a legendary panacea.

### Cell lines and culture

Cal27 cells were purchased from the American Tissue Culture Collection (ATCC, Cat# CRL-2095™). HN12 cells were a kind gift from Dr. Andrew Yeudall in 2016 and maintained in our lab. SCC1 and SCC15 cells were obtained courtesy of Dr. Georgia Chen at Emory University. MOC2 cells were obtained from Kerafast. All cell lines were cultured in DMEM/F12 medium (Gibco, Cat# 11320033) supplemented with 5% FBS (Hyclone, Cat# 16777-002) and 1% penicillin-streptomycin (Gibco, Cat# 15240062) and maintained at 37 °C in a humidified incubator with 5% CO_2_. Cells were used for experiments before passage 10 and were routinely screened for mycoplasma contamination by MycoAlert Mycoplasma Detection Kit (ATCC, Cat# 30–1012 K).

### Reagents

AZD5438 (Cat# HY-10012), PD0325901 (Cat# HY-10254), collagenase II (Cat# HY-E70005B) and Y-27632 (Cat# HY-10071) were purchased from MCE (Monmouth Junction, NJ). N-acetylcysteine (NAC) (Cat# A0150000) and 2’,7’-Dichlorofluorescein (DCF, Cat# 35848) were obtained from Sigma Aldrich.

### Drug IC_50_ determination and drug synergism

To measure the IC₅₀ of the drug, cells are plated in 96-well format and exposed in triplicate to a serial dilution of the drug. Following 72 h of drug incubation, cell viability is assessed using MTT (Sigma, Cat# 475989). Viability is expressed as a percentage relative to vehicle-treated controls, and the IC₅₀ value is obtained by fitting the data to a logistic dose-response curve. For drug synergy prediction, individual values of relative cell viability from each well were measured and analyzed using online SynergyFinder web application [23]. Synergy scores < −10, from − 10 to 10, and >10 indicate an antagonistic, additive, and synergistic effect, respectively.

### Cell colony formation

HNSCC cells were seeded into six-well plates at a density of 1 × 10³ cells per well and incubated at 37 °C in a humidified incubator with 5% CO₂. After 10 days of drug treatment, cells were fixed with 10% formaldehyde (Sigma, Cat# HT501128), stained with 0.4% crystal violet (Sigma, Cat# C0775), and colonies containing more than 50 cells were counted.

### Western blot

Whole-cell lysates were prepared in cell lysis buffer (Cell Signaling Technology, Cat# 9803) supplemented with protease inhibitors (Thermo Scientific, Cat# A32961). Protein concentrations were determined using the BCA Protein Assay (Thermo Fisher, Cat# 23225), and 30 µg of total protein per sample was resolved on 8% SDS-PAGE gels. Proteins were transferred to Immobilon PVDF membranes (Millipore, Cat# IPFL00010), which were then blocked in TBST containing 5% BSA (Thermo Scientific, Cat# AAJ65966-22) before incubation with the appropriate primary antibodies. Detection was performed using the Immobilon Western HRP substrate (Southern Biotechnology, Cat# 100242-504 and 100244-772), and chemiluminescent signals were visualized with an Amersham Imager 600 (GE). Specific antibodies used for western blot were ERK1/2 (Cell Signaling Technology, Cat# 4695), p-ERK1/2 (Thr202/Tyr204) (Cell Signaling Technology, Cat# 4370), Rb (Cell Signaling Technology, Cat# 9313), and p-Rb (Ser807/811) (Cell Signaling Technology, Cat# 8516), c-PARP (Cell Signaling Technology, Cat# 9544), and β-actin (Sigma Aldrich, Cat# A5316).

### BioID2-ER construct

An ER lumen-resident BioID expression plasmid (termed ER-BioID^HA^) was a kind gift from Dr. Toren Finkel at University of Pittsburgh [24], which was designed to contain the BioID coding sequence with the following features in order: IgK signal peptide, BioID2 coding sequence, HA tag, and the ER retention signal KDEL (Lys-Asp-Glu-Leu) tetrapeptide. To express ER-BioID^HA^ in HN12 cells (HN12-ER cells), cells were infected overnight with ER-BioID^HA^ lentivirus at a multiplicity of infection (MOI) of 10 as described previously [24, 25]. Stable HN12-ER cells were selected by 1.5 µg/ml puromycin (Thermo Scientific, Cat# J67236.8EQ) for one week.

### Liquid chromatography-mass spectrometry (LC-MS)

HN12-ER cells were pulsed with 50 µM biotin (MCE, Cat# HY-B0511) for 12 h as described previously [26]. After two washes with PBS to remove residual biotin in the media, cells were treated with or without 2 µM AZD5438 and 1 µM PD0325901 for 48 h. Supernatants were collected and incubated with streptavidin beads (Invitrogen, Cat# 65605D) overnight. Beads were retrieved using a magnetic stand and analyzed by mass spectrometry at the Taplin Mass Spectrometry Facility, Harvard Medical School. LC-MS data were processed with MaxQuant v2.5.2.0 software to generate peak lists for comprehensive database searching. Differentially secreted proteins were identified and compared between HN12 cells with or without the combination treatment (∣fold change∣ ≥ 2, *p* < 0.05). The resulting spectra were visualized in two-dimensional plots with retention time on the x-axis and m/z ratio on the y-axis.

### Apoptosis analysis

For in vitro analysis, apoptosis was determined by flow cytometry using the Annexin V: PE Apoptosis Detection Kit (Invitrogen, Carlsbad, CA, Cat# 88–8102-72). For in vivo analysis, apoptosis was assessed by staining for DNA fragmentation using In Situ Cell Death Detection Kit, TMR red (Roche, Cat# 12156792910). The average number of TUNEL-positive cells in each section in different treatments were counted in twenty randomly selected fields of view using a fluorescence microscope.

### Enzyme-linked immunosorbent assays (ELISA)

LDH release was measured using the Pierce™ LDH Cytotoxicity Assay Kit (Thermo Fisher Scientific, Cat# 88953) according to the manufacturer’s protocol. Relative LDH release was expressed as the percentage LDH activity in culture supernatants compared to total LDH (from media and cells) and used as an index of cytotoxicity. Extracellular ATP levels were determined using ATP Assay Kit (Abcam, Cat# ab83355). HMGB1 levels in culture supernatants were measured with HMGB1 ELISA kits (ABclonal, Cat# RK06736; Bio-techne, Cat# NBP2-62766).

### WGS and data processing

WGS was performed on genomic DNA of individual HNSCC PDOs by Novogene (QIAGEN, Cat# 56304). DNA libraries were prepared using the NEBnext DNA Library preparation kit and sequenced using the Illumina sequencing technology and 150 bp paired-end reads. Paired-end clean reads were aligned to the human reference genome (GRCh38.p14) using the Burrows-Wheeler Aligner (BWA). The initial alignment output was generated in BAM format. Post-alignment processing was performed using Sambamba [27] to sort BAM files and mark duplicate reads.

### Animal study

Six-week-old male NOD.Cg-*Prkdcscid Il2rgtm1Wjl/SzJ* (NSG) (Cat# 005557), B6.129S2-*Tcra*^tm1Mom^/J (*Tcra* KO) (Cat# 002116) and wildtype C57BL/6 (Cat# 000664) mice were purchased from the Jackson Laboratory (Bar Harbor, ME). All animal experiments were approved by the Institutional Animal Care and Use Committee (IACUC) of Emory University. To evaluate the antitumor efficacy of the drugs in human HNSCC, an orthotopic tumor mouse model was generated by injection of 5 × 10^5^ HN12 cells into the buccal mucosa of NSG mice. Ten days after tumor cell injection (~ 100 mm^3^ in tumor volume), mice were randomly assigned to receive vehicle (corn oil), PD0325901, AZD5438, or both drugs in combination. AZD5438 was dissolved in corn oil and given by oral gavage at 50 mg/kg once daily for 14 days. PD0325901 was dissolved in corn oil with 5% DMSO and given at 25 mg/kg once daily for 14 days. For the combination group, both drugs were mixed and given together by oral gavage once daily for 14 days. To assess the therapeutic benefit of the nanodrugs, NSG mice bearing orthotopic HN12 tumors were randomized to receive vehicle, the combination of AZD5438 and PD0325901, or Nano-(AZD + PD). Nano-(AZD + PD) was administered intratumorally at a volume of 100 µL, containing 0.058 mg/mL AZD5438 and 0.03 mg/mL PD0325901 in a 2:1 weight ratio, every other day for a total of seven doses. Vehicle and the free drug combination was delivered using the same treatment regimen. To assess T cell-mediated immune response to the combination of AZD5438 and PD0325901, an orthotopic synergetic mouse model was established by injection of 5 × 10^5^ MOC2 cells into the buccal mucosa of *Tcra* KO or wildtype C57BL/6 mice. Ten days after tumor cell inoculation, mice were randomly assigned to receive vehicle, Nano-(AZD + PD), or the free drug combination by intratumoral injection. Treatment dose and procedure were the same to those used in NSG mice. Tumor dimensions were serially measured with electronic calipers, and tumor volume was calculated by the formula of V = length × width^2^ × 1/2. Body weight and physical activity of each animal were followed as markers of toxicity. Afterwards, mice were sacrificed, and tumors and major organs (including the heart, intestine, kidney, liver, lung and spleen) were excised for histopathological and flow cytometry analysis.

### Tumor dissociation and organoid culture

Tumor tissues from HNSCC patients were excised and placed in DMEM/F12 medium on ice. Following three washes with ice-cold DPBS containing 100 µg/ml Primocin (InvivoGen, Cat# ant-pm-05), tumor tissues were minced into approximately 2 mm fragments, followed by incubated in DMEM/F12 medium supplemented with 5 mg/ml collagenase II and 10 µM Y-27,632 for 1 h at 37 °C. After centrifugation and removal of the supernatant, 1 ml of TrypLE (Thermo Fisher Scientific, Cat# 12–605-010) with 10 µM Y-27,632 were added, and the samples were incubated at 37 °C for 10 min. After filtration through a 70 μm strainer, cells were seeded at 3 × 10⁵ cells/mL in OncoPro Tumoroid Culture Medium (Thermo Fisher, Cat# A5701201) with 10 µM Y-27,632 and mixed with 2% v/v Matrigel (Corning, Cat# 356234). For tumoroid formation, 50 µl domes containing 1 × 10⁵ viable cells with 10 mg/ml Matrigel were plated, polymerized at 37 °C for 30 min, and overlaid with supplemented OncoPro Tumoroid Culture Medium. Medium was refreshed every three days, and tumoroids were harvested for analysis upon reaching ~ 200 μm in diameter, which was measured using ImageJ. To detect the degree of cell death in organoids, organoid medium was supplemented with Hoechst 33,342 (10 µg/ml) (Invitrogen, Cat# H21486), Calcein AM (5 µM) (Invitrogen, Cat# C3100MP) and PI (10 µg/ml) (Invitrogen, Cat# P1304MP) and incubated for 1 h under the growth condition. Organoids were then imaged on an Echo Revolve fluorescence microscope. The intensity of each organoid’s PI was measured with ImageJ.

### Flow cytometry

Tumors excised from C57BL/6 mice treated with vehicle, the combination of AZD5438 and PD0325901, or Nano-(AZD + PD) were digested in X-Vivo-15 (Lonza, Cat# 02-053Q) supplemented with Collagenase Type 4 (Worthington Biochemical, Cat# LS004188) and DNase (Roche, Cat# 03724778103) and incubated at 37 °C, 5% CO2 for 30 min before being filtered through a 70 μm cell strainer. Single-cell suspensions from tumors were rinsed in FACS buffer (Biolegend, Cat# 420201), then stained with surface antibodies for 30 min, followed by additional rinsing and fixation (Biolegend, Cat# 420801). UltraComp eBeads™ Plus Compensation Beads (Cat# 01–3333-42) and LIVE/DEAD™ Fixable Aqua Dead Cell Stain Kit (Cat# L34957) were purchased from Invitrogen. The following fluorochrome-conjugated antibodies specific to murine were used for flow cytometry analysis: CD3 (17A2, BD Biosciences, Cat# 560527), CD8 (53 − 6.7, BioLegend, Cat# 100722), CD45 (30-F11, BioLegend, Cat# 103116), granzyme B (GB11, BioLegend, Cat# 515406), and IFNγ (XMG1.2, BioLegend, Cat# 505810). Samples were run on a BD Symphony A3 cytometer and data were analyzed using FlowJo (V.10.8.1) software (BD Biosciences). ‘Fluorescence minus one’ controls were tested for each multicolor flow panel.

### Immunohistochemistry (IHC)

Tissue sections were deparaffinized in xylene (Avantor, Cat# 89370-088), rehydrated through a graded series of alcohol (Avantor, Cat# 89370-084) and incubated in 3% hydrogen peroxide (Sigma, Cat# 323381). Sections were placed in 10 mM sodium citrate buffer (pH 6.0) (Fisher, Cat# BP327) at sub-boiling temperatures for 10 min and incubated with 10% normal goat serum (Jackson immunoresearch, Cat# 005-000-121), followed by incubation with primary antibodies. Immunoreactivity was visualized using the DAB Detection kit (Vector Laboratories, Burlingame, CA, Cat# SK-4105) and counterstained with hematoxylin. Slides were then dehydrated, mounted, and scanned using an Olympus Nanozoomer whole slide scanner (Olympus, Center Valley, PA). At least 10 random microscopic fields were captured per sample, and signal intensity was semi-quantified using ImageJ Fiji (version 1.2). Specific antibodies used for IHC were p-Rb (Cell Signaling Technology, Cat# 8516), p-ERK1/2 (Cell Signaling Technology, Cat# 4370), CD8 (Cell Signaling Technology, Cat# 98941), and Ki67 (Sigma Aldrich, Cat# SAB5600249).

### Lipid synthesis and fabrication of LNP formulations

Polyamine-containing ionizable lipid 113-O14O was synthesized, purified, and characterized following our previously reported procedures [28]. Cholesterol was purchased from Sigma, and DOPC and DMG-PEG2000 were purchased from Avanti Polar Lipids. 113-O14O, cholesterol, DOPC, and DMG-PEG2000 were mixed in ethanol with a weight ratio of 16.5/4/2/1. AZD5438 and PD0325901 were dissolved in DMSO and mixed with the lipid ethanol solution. Under vigorous vertexing, the organic solution was added to PBS dropwise. The solution was stirred for another ten minutes before dialysis (Thermo Fisher, Slide-A-Lyzer).

### Characterization of LNP formulations

LNP formulations were characterized following the methods described in our previous study [29]. Average hydrodynamic diameter (< *D*_h_>) and PDI of LNP formulation were measured by Malvern ZetaSizer Ultra at room temperature. CryoEM images were taken on a cryo transmission electron microscope (JEOL JEM-2100). LNP solution was vitrified on Au-Flat 1.2/1.3 holey grids (Protochips) using a Leica EM GP2 cryo plunger (6 °C, 85% humidity, five seconds blot time). Grids were mounted on a Gatan 914 side entry cryo specimen holder and imaged in a JEOL JEM-2100 F transmission electron microscope operating at 200 kV. Encapsulation efficacy of AZD5438 and PD0325901 and their release profile were measured using HPLC system (Waters) equipped with 1525 Binary Pump and 2489 UV/Visible Detector. HPLC grade acetonitrile and water (VWR) were used as the mobile phase.

### Statistical analysis

Statistical analysis was performed with statistical software GraphPad Prism 9 (San Diego, CA). All central tendencies indicate the mean, and all error bars indicate SD. Survival curves were drawn using the Kaplan-Meier method, and the differences between the two curves were compared with the log-rank Mantel-Cox test. Statistical significance was determined using Student’s t-test or two-way ANOVA with post-hoc Tukey analysis. Differences were considered statistically significant when *p* < 0.05.

## Results

### AZD5438 has strong potential to be combined with PD0325901 for the treatment of HPV (−) HNSCC with high mutational burden

**Fig. 1:**
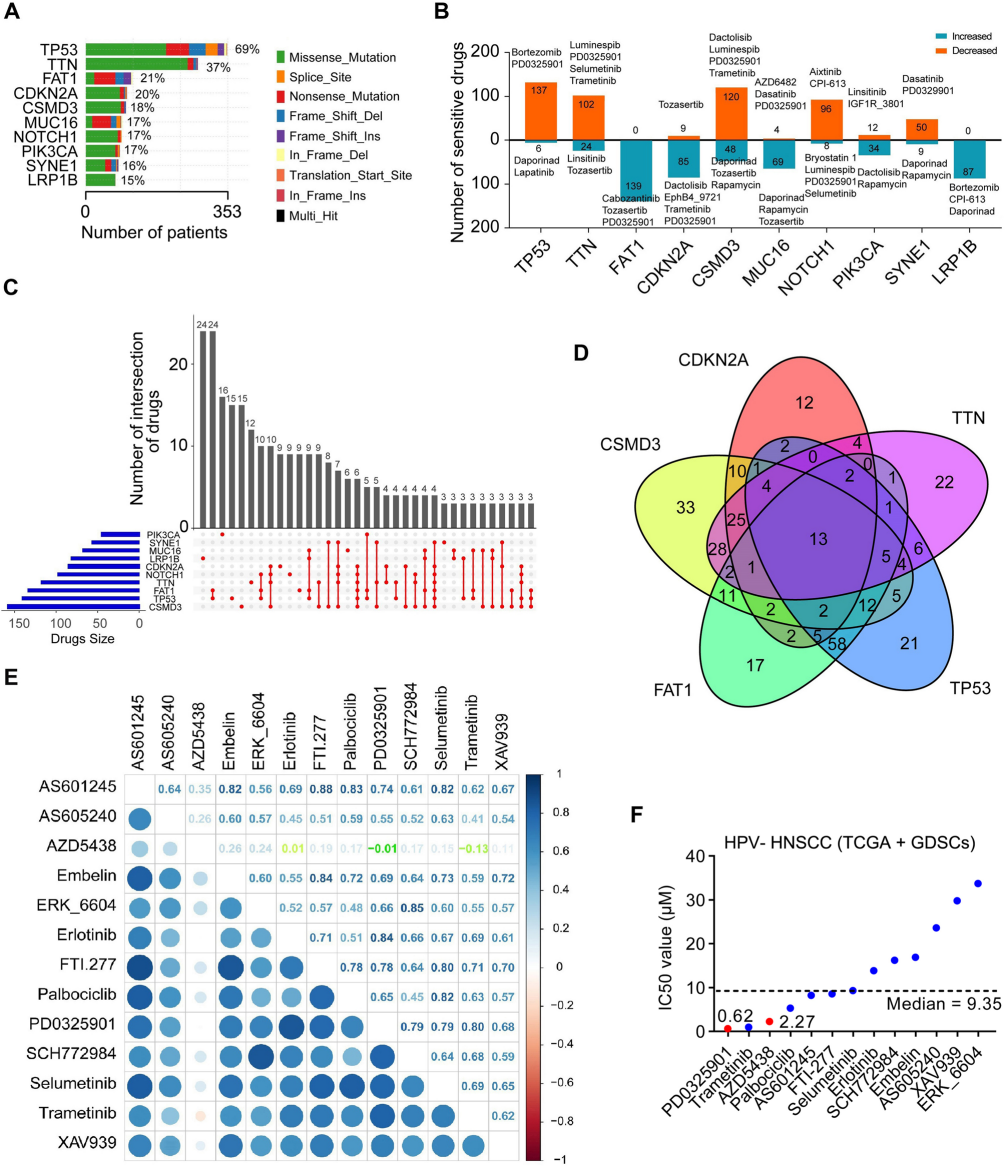
Drug sensitivity analysis of HPV (−) HNSCC cases with the most frequently mutated genes using the combined data from TCGA and GDSC datasets. **A** Gene mutation analysis for HPV (−) HNSCC in TCGA database. The top 10 most frequently mutated genes are shown with the corresponding mutation rate. **B** Drug sensitivity associated with the expression of the top 10 most frequently mutated genes in HPV (−) HNSCC patients using TCGA and GDSC datasets. The number of drugs resistant or sensitive to each mutated gene and representative drugs are shown. **C** Upset plots showing the number of drug intersections among the top 10 most frequently mutated genes based on drug sensitivity analysis. **D** Venn plots showing the number of drug intersections among the top 5 most frequently mutated genes (*TP53*, *TTN*, *FAT1*, *CDKN2A* and *CSMD3*) based on drug sensitivity analysis. **E** Correlation assessment of 13 common drugs that are sensitive to the top 5 most frequently mutated genes. This analysis is based on the IC_50_ of each drug. **F** IC_50_ of 13 common drugs that are sensitive to top 5 most frequently mutated genes in HPV (−) HNSCC patients

Based on TCGA database, we identified that *TP53* (69%), *TTN* (37%), *FAT1* (21%), *CDKN2A* (20%), *CSMD3* (18%), *MUC16* (17%), *NOTCH1* (17%), *PIK3CA* (17%), *SYNE1* (16%) and *LRP1B* (15%) are the top 10 most frequently mutated genes in the HPV (−) HNSCC cohort (Fig. 1A). Given that cell line drug screening datasets such as GDSC1 and GDSC2 are widely used in drug discovery applications [22], we integrated TCGA and GDSC HNSCC datasets to generate drug response matrices, enabling the prediction of clinical drug responses in patients harboring the specified gene mutations. Using the OncoPredict R package, we identified drugs whose predicted sensitivity was associated with the expression of the top 10 most frequently mutated genes in HPV (−) HNSCC patients (Fig. 1B). A total of 139 drugs, including cabozantinib and PD0325901, were predicted to be more effective in HPV (−) HNSCC cases characterized by high expression of mutated *FAT1* (Fig. 1B). PD0325901 was also predicted to be more effective in HPV (−) HNSCC cases with high expression of mutated *CDKN2A* or *NOTCH1* (Fig. 1B).

Next, the UpSetR package was employed to consolidate the drugs exhibiting significant differential sensitivity across the top 10 most frequently mutated genes. This analysis revealed the number of drugs uniquely associated with individual mutated genes (Fig. 1C). For example, 15 drugs showed sensitivity uniquely associated with *TP53* gene mutations, while 9 drugs showed sensitivity uniquely associated with *FAT1* gene mutations (Fig. 1C). Venn plots revealed 13 drugs (PD0325901, AZD5438, Trametinib, Palbociclib, AS601245, FTI-277, Selumetinib, Erlotinib, SCH772984, ERK_6604, Rmbelin, AS605240 and XAV939) whose sensitivity was commonly associated with the top 5 most frequently mutated genes (*TP53*, *TTN*, *FAT1*, *CDKN2A*, and *CSMD3*) (Fig. 1D). Notably, drug correlation analysis indicated that the CF between PD0325901 and AZD5438, a potent oral inhibitor of multiple cyclin-dependent kinases, was − 0.01, the lowest CF observed between PD0325901 and any of the 13 drugs identified (Fig. 1E). The lack of strong association between PD0325901 and AZD5438 implies complementary mechanisms, suggesting that their combination could achieve strong synergistic efficacy. Moreover, drug IC_50_ data in the GDSC HNSCC cell line datasets demonstrated that PD0325901 and AZD5438 ranked among the top three most sensitive drugs out of the 13 drugs identified in HPV (−) HNSCC (Fig. 1F). Taken together, our bioinformatics analysis suggests that the combination of AZD5438 and PD0325901 may represent a promising treatment strategy for HPV (−) HNSCC with high mutational burden.

### The combination of AZD5438 and PD0325901 exhibits superior efficacy in suppressing HNSCC with high mutational burden compared to either treatment alone

**Fig. 2:**
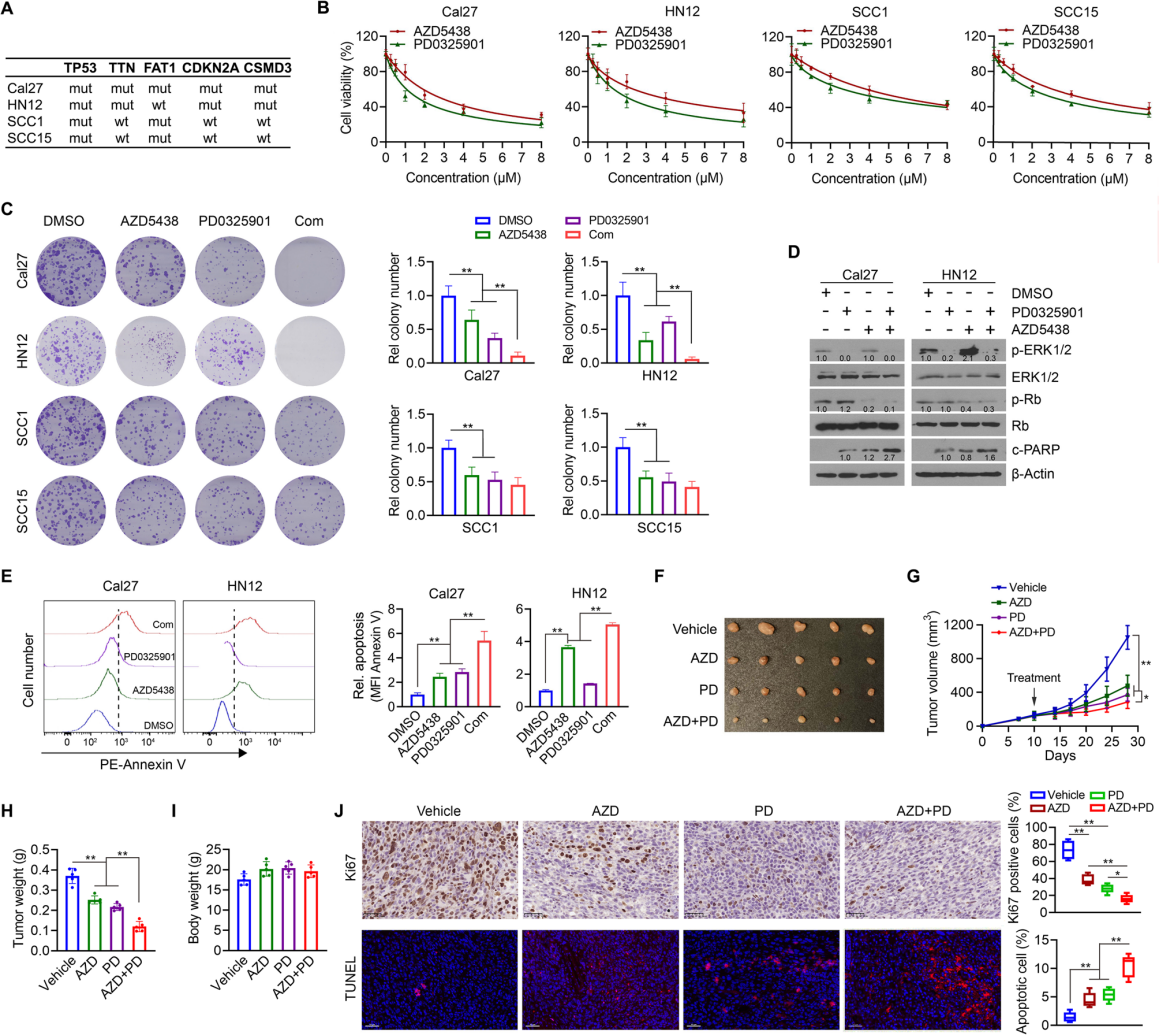
Synergistic antitumor activity of AZD5438 in combination with PD0325901 in HNSCC. **A** A table summarizing the mutation status of the five most commonly mutated genes in HPV (−) HNSCC across four HNSCC cell lines. **B** IC_50_ of AZD5438 and PD0325901 determined in four HNSCC cell lines (*n* = 3 biological replicates/dose). HNSCC cells were treated with the drug for 72 h before analysis. **C** Effect of AZD5438 and/or PD0325901 treatment on clonogenic ability of four HNSCC cell lines. HNSCC cells were treated with the drug for 10 days before analysis. Quantitative data from 3 biological replicates are shown in the right panel. **D** Effect of AZD5438 and/or PD0325901 treatment on the phosphorylation levels of ERK1/2 and Rb and c-PARP cleavage in Cal27 and HN12 cells (*n* = 3 biological replicates). HNSCC cells were treated with the drug for 48 h before analysis. **E** Effect of AZD5438 and/or PD0325901 treatment on apoptosis of Cal27 and HN12 cells determined by Annexin V staining (*n* = 3 biological replicates). HNSCC cells were treated with the drug for 48 h before analysis. **F-I** Tumor images, growth curve and weight and mouse body weight in different treatment groups (*n* = 5 mice/group). An orthotopic HN12 tumor model was established in the buccal mucosa of NSG mice, followed by oral administration of vehicle, PD0325901, AZD5438, or their combination once daily for 14 days. Tumors were excised on Day 28 after cell inoculation for IHC analysis. **J** Immunostaining of Ki67 and TUNEL assays for tumor tissues derived from the groups treated with vehicle, AZD5438, PD0325901, or the AZD5438 and PD0325901 combination. Representative results and quantitative data (*n* = 5 independent samples) are shown in the left and right panels, respectively. Data are presented as mean values +/- SD. For statistical comparisons, *p* values were assessed by unpaired, two-tailed Student’s t test. *p < 0.05; **p < 0.01

HN12 and Cal27 cells harbor mutations in four or five of the five most mutated genes in HPV (−) HNSCC, respectively (Fig. 2A). In contrast, SCC1 and SCC15 cells exhibit mutations in only two of these five frequently altered genes (Fig. 2A). The IC₅₀ values of AZD5438 in HN12, Cal27, SCC1, and SCC15 cells were 3.852, 2.988, 5.881, and 4.787 µM, respectively (Fig. 2B and Table S1). The IC₅₀ values of PD0325901 in these cell lines were 2.070, 1.729, 4.712, and 3.229 µM, respectively (Fig. 2B and Table S1). Notably, compared to SCC1 and SCC15 cells, Cal27 and HN12 cells displayed lower IC_50_ for both AZD5438 and PD0325901. To determine whether AZD5438 and PD0325901 exert synergistic inhibitory effects on HNSCC cells, we analyzed drug interactions in Cal27, HN12, SCC1, and SCC15 cells by SynergyFinder. This drug combination demonstrated a strong synergistic inhibitory effect in Cal27 and HN12 cells, with synergy scores exceeding 10 (Fig. S1). In contrast, the combination exhibited only an additive effect in SCC1 and SCC15 cells, as indicated by synergy scores between − 10 and 10 (Fig. S1). Consistently, colony formation assays conducted at drug concentrations corresponding to IC_50_ demonstrated much stronger inhibitory effect of AZD5438 in combination with PD0325901 in Cal27 and HN12 cells, but not in SCC1 and SCC15 cells (Fig. 2C). These data suggest that the combination of AZD5438 and PD0325901 may selectively exert synergy in suppressing the growth of HNSCC with high mutation rates. At the molecular level, the combination of AZD5438 and PD0325901 effectively reduced the phosphorylation of both ERK1/2 and Rb, leading to increased apoptosis compared to either treatment alone in Cal27 and HN12 cells (Fig. 2D and E and Fig. S2-S3).

To assess the antitumor activity, HN12 tumor-bearing NSG mice were treated with vehicle, AZD5438, PD0325901, and the drug combination, respectively. Two weeks after cell inoculation, tumor growth and weight were significantly reduced in single-agent treatment groups compared to control group (Fig. 2F and H). Notably, the combination treatment led to a more pronounced reduction in tumor burden, highlighting its superior antitumor efficacy (Fig. 2F and H). None of the treatments, alone or in combination, affected the body weight of tumor-bearing mice during the experiment period (Fig. 2I). Moreover, tumors treated with the combination therapy showed a reduced number of Ki67-positive proliferating cells and an increased number of apoptotic cells compared to single-agent or control treatments (Fig. 2J). These findings support the notion that AZD5438 can enhance the therapeutic efficacy of PD0325901, leading to improved antitumor activity.

### Co-delivery of AZD5438 and PD0325901 using LNPs maximizes the drug synergistic antitumor effect

**Fig. 3:**
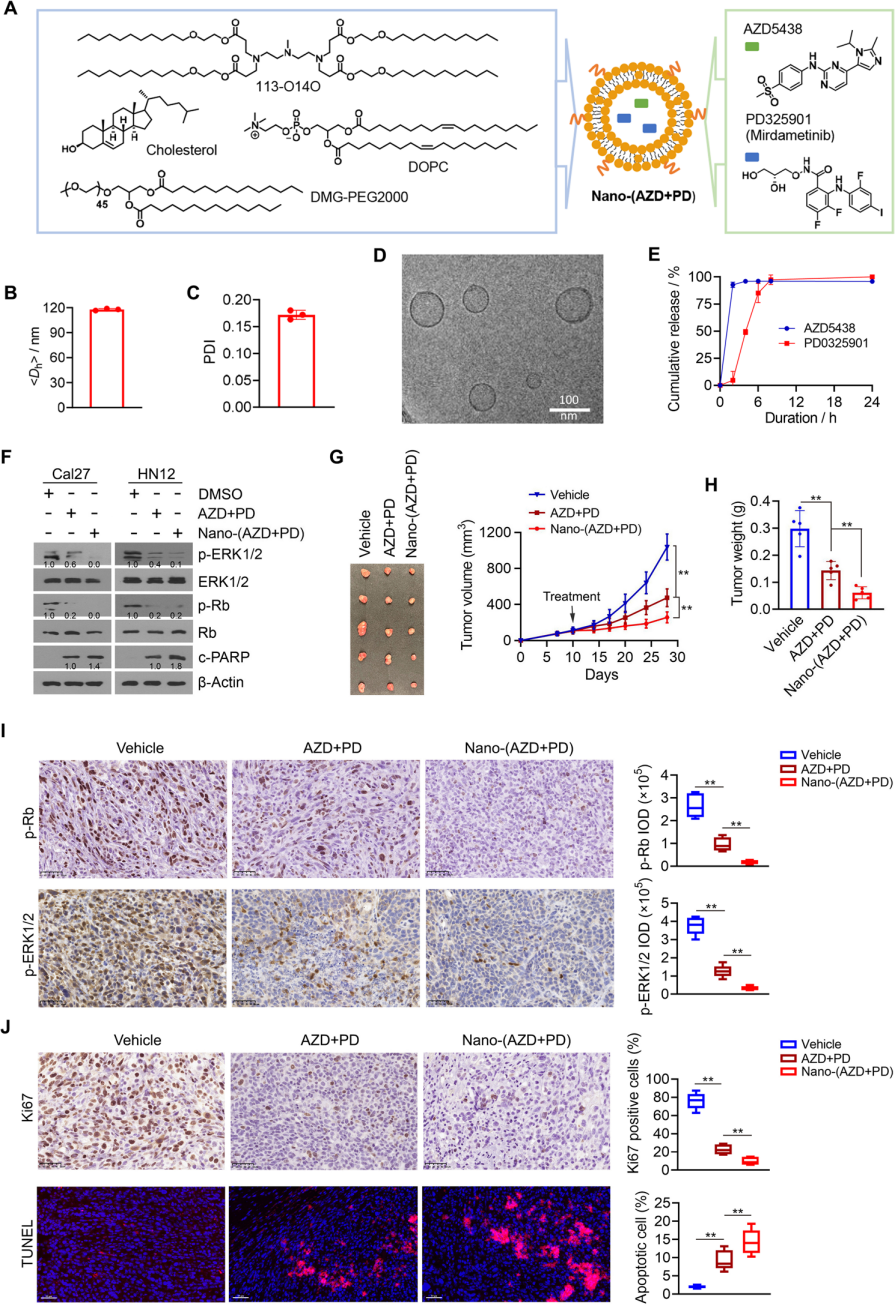
Nano-(AZD + PD) achieves a superior antitumor effect than the free drug combination in orthotopic xenograft mice. **A** Schematic illustrating the chemical compositions of active lipid (113-O14O), cholesterol, DOPC, and DMG-PEG2000 within the Nano-(AZD + PD) formulation. **B** Hydrodynamic size of Nano-(AZD + PD). **C** PDI of Nano-(AZD + PD). **D** Representative cryoEM image of Nano-(AZD + PD). **E** Drug release profile of Nano-(AZD + PD) at PH = 7.0. **F** Effect of Nano-(AZD + PD) and the free drug combination on targeting pathways in Cal27 and HN12 cells (*n* = 3 biological replicates). HNSCC cells were exposed to the various treatments for 48 h before analysis. **G**,** H** Tumor images, growth curve and weight in different treatment groups (*n* = 5 mice/group). An orthotopic HN12 tumor model was established in the buccal mucosa of NSG mice, followed by intratumoral administration of vehicle, the free drug combination, or Nano-(AZD + PD) every other day for a total of seven doses. Tumors were excised on Day 28 after cell inoculation for IHC and TUNEL analysis. **I** Immunostaining of phospho-Rb (p-Rb) and phospho-ERK1/2 (p-ERK1/2) using tumor tissues from mice treated with vehicle, the free drug combination, or Nano-(AZD + PD). **J** Immunostaining of Ki67 and TUNEL assays using tumor tissues from mice treated with vehicle, the free drug combination, or Nano-(AZD + PD). In (**I**) and (**J**), Representative results and quantitative data (*n* = 5 independent samples) are shown in the left and right panels, respectively. Data are presented as mean values +/- SD. For statistical comparisons, *p* values were assessed by unpaired, two-tailed Student’s t test *p < 0.05; **p < 0.01

A LNP formulation encapsulating AZD5438 and PD0325901, termed Nano-(AZD + PD), was synthesized using a self-assembly approach (Fig. 3A). Based on the IC_50_ of AZD5438 and PD0325901 determined in HN12 cells (Fig. 2B), the two drugs were combined in the LNP formulation at an approximate 2:1 weight ratio. This ratio was selected to optimize the therapeutic efficacy of the combination while maintaining a balanced and synergistic drug delivery profile. The physicochemical properties of Nano-(AZD + PD) were characterized using dynamic light scattering (DLS) and cryo-electron microscopy (cryoEM). The formulation displayed an average hydrodynamic diameter (< *D*_h_>) of 117.9 nm (Fig. 3B), making it well-suited for intracellular delivery [30, 31]. The polydispersity index (PDI) of Nano-(AZD + PD) was measured at 0.17, indicating a high degree of monodispersity (Fig. 3C). CryoEM analysis revealed that Nano-(AZD + PD) formed unilamellar vesicles (liposomes), with sizes consistent with those identified from DLS measurements (Fig. 3D). High-performance liquid chromatography (HPLC) was used to determine the drug loading content (DLC), revealing that AZD5438 and PD0325901 were loaded at 5.8% and 3.0%, respectively. In vitro drug release studies in PBS at 37 °C showed that both AZD5438 and PD0325901 were nearly completely released (96.1% and 97.4%, respectively) within 8 h (Fig. 3E). Although in vitro conditions may not fully replicate the in vivo environments, the rapid release profile is anticipated to facilitate swift drug action and therapeutic response following intratumoral injection.

Next, we evaluated the in vitro on-target effects of Nano-(AZD + PD) to determine its ability to modulate downstream signaling pathways. Similar to the effects observed with the free-drug combination, treatment with Nano-(AZD + PD) led to a reduction in both phosphorylated ERK1/2 and Rb levels and an increase in cleaved PARP (Fig. 3F and Fig. S2). In orthotopic tumor mouse models, Nano-(AZD + PD) demonstrated superior antitumor activity, leading to a significantly greater reduction in tumor growth compared to the free drug combination (Fig. 3G and H). H&E staining revealed no morphological changes in the major organs (Fig. S4), suggesting that this combined drug treatment, with or without LNP encapsulation, does not produce significant systemic toxicity during the study period. IHC analysis further showed that tumors from mice treated with Nano-(AZD + PD) displayed significantly reduced levels of phospho-ERK1/2 and phospho-Rb, along with a marked decrease in Ki67-positive proliferating cells, compared to those treated with the free drug combination (Fig. 3I and J). Furthermore, TUNEL assays revealed that Nano-(AZD + PD) induced a more robust apoptotic response, as indicated by a higher number of TUNEL-positive cells in the Nano-(AZD + PD) treatment group (Fig. 3J). Collectively, these findings provide strong preclinical evidence that LNP-mediated co-delivery of AZD5438 and PD0325901 enhances therapeutic efficacy in HNSCC.

### PD0325901 potentiates AZD5438-induced pyroptosis in HNSCC cells by activating caspase-8/gasdermin E (GSDME) signaling pathway

**Fig. 4:**
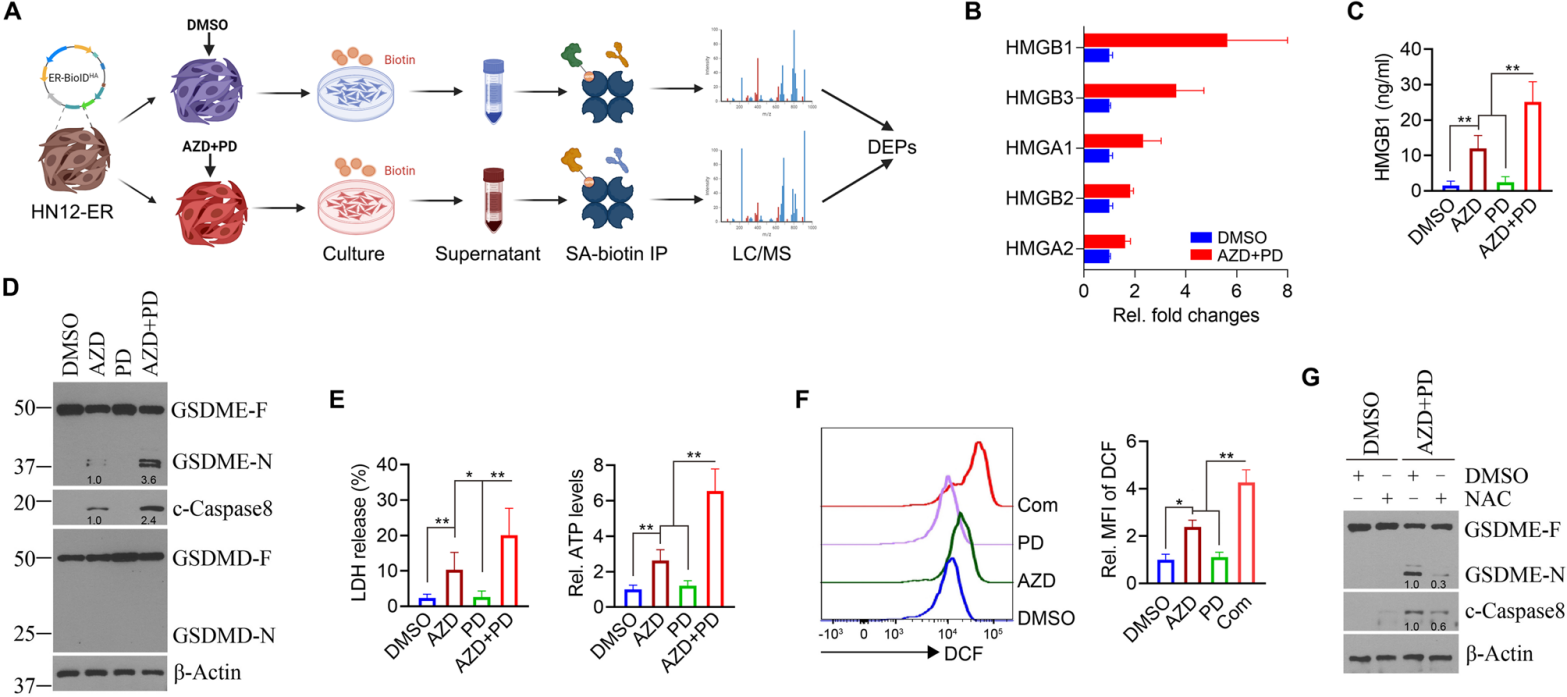
PD0325901 enhances AZD5438-induced pyroptosis in HNSCC cells. **A** Schematic depicting the workflow of BioID-based secretome analysis to determine alterations in proteins secreted by HN12 cells in the presence or absence of the combination of AZD5438 and PD0325901. Figure created using Biorender (https://biorender.com). **B** Fold changes in HMGB family members secreted from HN12-ER cells treated with or without the combination of AZD5438 and PD0325901 for 48 h, determined by mass spectrometric analysis (*n* = 2 biological replicates). **C** Effect of AZD5438 and/or PD0325901 on HMGB1 levels in HN12 cell supernatants determined by ELISA (*n* = 6 independent samples). HN12 cells were exposed to the various treatments for 48 h before analysis. **D** Effect of AZD5438 and/or PD0325901 on the activation of pyroptotic pathway in HN12 cells (*n* = 3 biological replicates). HN12 cells were exposed to the various treatments for 48 h before analysis. **E** Effect of AZD5438 and/or PD0325901 on LDH and ATP levels in HN12 cell supernatants determined by ELISA (*n* = 6 independent samples). HN12 cells were exposed to the various treatments for 48 h before analysis. **F** Effect of AZD5438 and/or PD0325901 on ROS levels in HN12 cells determined by flow cytometry (*n* = 3 independent samples). HN12 cells were exposed to the various treatments for 48 h before analysis. **G** Effect of NAC on the activation of the pyroptotic pathway induced by the combination of AZD5438 and PD0325901 in HN12 cells (*n* = 3 biological replicates). HN12 cells were pretreated with 10 mM NAC for 24 h, followed by a 48-hour exposure to the combination of AZD5438 and PD0325901. Data are presented as mean values +/- SD. For statistical comparisons, *p* values were assessed by unpaired, two-tailed Student’s t test. *p < 0.05; **p < 0.01

To elucidate the molecular mechanisms driving the synergistic antitumor effects of AZD5438 and PD0325901 in HNSCC cells, we performed a proximity biotinylation-based secretome analysis following our previously established protocol [25, 26]. This analysis revealed significant alterations in the protein secretion profile of HN12 cells treated with the drug combination compared to controls (Fig. 4A). Notably, the combination treatment led to increased protein secretion of high mobility group (HMG) superfamily members, including HMGB1, HMGB2, HMGB3, HMGA1, and HMGA2, in the supernatant from HN12 cells (Fig. 4B). Among them, HMGB1, a key downstream marker of pyroptosis [25, 32], was the most significantly elevated following combination treatment. ELISA analysis of the HN12 cell supernatants confirmed that while AZD5438 alone increased HMGB1 levels, the addition of PD0325901 further enhanced its secretion (Fig. 4C). Moreover, the formation of cell membrane pores and membrane rupture, distinct morphological features of pyroptosis, were observed in both HN12 and Cal27 cells following AZD5438 treatment (Fig. S5A). These morphological changes were even more pronounced with the addition of PD0325901 (Fig. S5A). We then examined the activation of the pyroptotic pathway in HN12 cells under single and combination drug treatments. AZD5438 alone induced the cleavage of caspase-8 and GSDME, while the combination of AZD5438 and PD0325901 enhanced pyroptosis more effectively, as evidenced by increased levels of cleaved caspase-8 and GSDME (Fig. 4D). Importantly, no activation of gasdermin D (GSDMD) was observed under either single or combined treatment conditions (Fig. 4D), suggesting that pyroptosis proceeded via a non-canonical pathway in this context. To assess cellular damage, we measured lactate dehydrogenase (LDH) release and ATP levels in the supernatants. As shown in Fig. 4E, the combination treatment led to significantly higher LDH release and increased extracellular ATP levels, indicating increased cell death and metabolic disruption associated with pyroptosis. Notable, flow cytometry analysis revealed that the combination treatment elevated reactive oxygen species (ROS) levels in HN12 cells (Fig. 4F and Fig. S3). To determine the role of ROS in pyroptosis activation, HN12 cells were pretreated with 10 mM of the antioxidant NAC for 24 h before being subjected to the combination treatment with AZD5438 and PD0325901. NAC significantly reduced pyroptotic activation (Fig. 4G), highlighting ROS as a key mediator of the drug combined effect. These results indicate that PD0325901 has a great potential to enhance AZD5438-induced pyroptosis in HNSCC cells.

### Nano-(AZD + PD) enhances antitumor efficacy by promoting pyroptosis-induced antitumor immunity

**Fig. 5:**
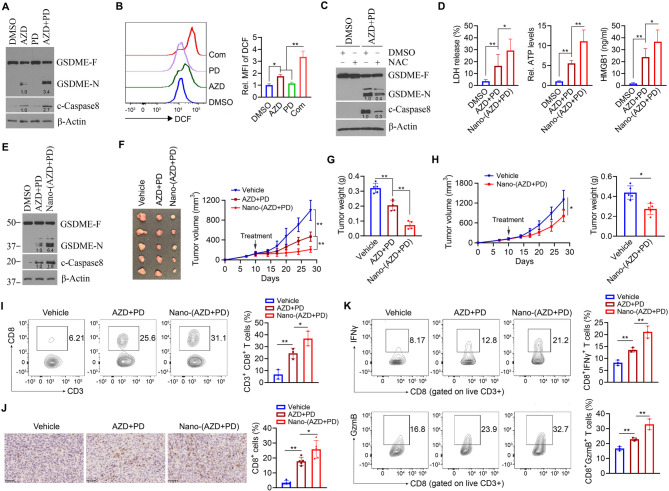
Nano-(AZD + PD) potentiates CD8^+^ T cell-mediated antitumor immunity in orthotopic syngeneic mice. **A** Effect of AZD5438 and/or PD0325901 on the activation of pyroptotic pathway in MOC2 cells (*n* = 3 biological replicates). MOC2 cells were exposed to the various treatments for 48 h before analysis. **B** Effect of AZD5438 and/or PD0325901 on ROS levels in MOC2 cells determined by flow cytometry (*n* = 3 independent samples). MOC12 cells were exposed to the various treatments for 48 h before analysis. **C** Effect of NAC on the activation of the pyroptotic pathway induced by the combination of AZD5438 and PD0325901 in MOC2 cells (*n* = 3 biological replicates). MOC2 cells were pretreated with 10 mM NAC for 24 h, followed by a 48-hour exposure to the combination of AZD5438 and PD0325901. **D** Effect of AZD5438 and/or PD0325901 on LDH, ATP and HMGB1 levels in MOC2 cell supernatants determined by ELISA (*n* = 6 independent samples). MOC12 cells were exposed to the various treatments for 48 h before analysis. **E** Effect of Nano-(AZD + PD) and the free drug combination on the activation of pyroptotic pathway in MOC2 cells (*n* = 3 biological replicates). MOC12 cells were exposed to the various treatments for 48 h before analysis. **F**,** G** Tumor images, growth curve and weight of C57BL/6 mice receiving different treatments (*n* = 5 mice/group). **H** Tumor growth curve and weight of *Tcra* KO C57BL/6 mice receiving different treatments (*n* = 5 mice/group). In (**F**-**H**), an orthotopic MOC2 tumor model was established in the buccal mucosa of wildtype (**F**, **G**) or *Tcra* KO (**H**) C57BL/6 mice, followed by intratumoral administration of vehicle, the free drug combination, or Nano-(AZD + PD) every other day for a total of seven doses. Tumors were excised on Day 28 after cell inoculation for IHC and flow cytometry analysis. **I** Percent of CD8^+^ T cell subset in MOC2 tumors receiving vehicle, the free drug combination of AZD5438 and PD0325901, or Nano-(AZD + PD) (*n* = 3 mice/group). **J** IHC for CD8 in MOC2 tumors receiving vehicle, the free drug combination of AZD5438 and PD0325901, or Nano-(AZD + PD) (*n* = 5 independent samples). **K** Percent of cytotoxic (GzmB^+^ and IFNγ^+^) CD8^+^ T cell subsets in MOC2 tumors receiving vehicle, the free drug combination of AZD5438 and PD0325901, or Nano-(AZD + PD) (*n* = 3 mice/group). Data are presented as mean values +/- SD. For statistical comparisons, *p* values were assessed by unpaired, two-tailed Student’s t test. *p < 0.05; **p < 0.01

Given that pyroptosis is a form of immunogenic cell death, we sought to investigate the impact of AZD5438 and PD0325901 on antitumor immune responses using a syngeneic mouse model. The mouse HNSCC cell line MOC2 carries mutations in *Trp53*, *Ttn*, *Fat1*, and *Csmd3* [33], representing four of the five most commonly mutated genes in HPV (−) HNSCC. We then treated MOC2 cells with either single agents or the combination of AZD5438 and PD0325901. Consistent with our findings in HN12 cells, AZD5438 alone activated the ROS-associated caspase-8/GSDME pyroptotic pathway, while the combination treatment further enhanced pyroptotic activation, as indicated by increased levels of cleaved caspase-8 and GSDME (Fig. 5A and C). Our results also showed that this combination significantly increased LDH release and HMGB1 secretion from MOC2 cells compared to either drug alone. Additionally, extracellular ATP levels were markedly elevated following combination treatment (Fig. 5D), further supporting the notion that the dual drug treatment promotes a robust pyroptotic response.

Next, we examined whether the nano-formulated co-delivery system, Nano-(AZD + PD), could further enhance pyroptotic activation. Compared to the free drug combination, Nano-(AZD + PD) induced more robust cleavage of caspase-8 and GSDME (Fig. 5E), resulting in more pronounced morphological features of pyroptosis (Fig. S5B). To evaluate its in vivo antitumor efficacy, we established an orthotopic MOC2 tumor model by implanting tumor cells into the buccal mucosa of immunocompetent C57BL/6 mice. Notably, Nano-(AZD + PD) led to significantly greater tumor suppression compared to the free drug combination (Fig. 5F and G). To investigate the involvement of the immune system in the enhanced antitumor effect, we repeated the experiment using T cell-deficient *Tcra* KO C57BL/6 mice. Compared to wildtype C57BL/6 mice, the therapeutic efficacy of Nano-(AZD + PD) was significantly reduced in *Tcra* KO mice (Fig. 5H), indicating that the improved antitumor response is at least partially dependent on T cell-mediated mechanisms.

To understand whether Nano-(AZD + PD) enhanced CD8^+^ T cell-mediated antitumor immunity, we analyzed tumor-infiltrating CD8^+^ T cells in MOC2 tumors following the treatment. Flow cytometry analysis revealed a significant increase in the proportion of CD8^+^ T cells in tumors treated with Nano-(AZD + PD) compared to those treated with the free drug combination or vehicle control (Fig. 5I and Fig. S3). This observation was further validated by IHC using an anti-CD8 antibody (Fig. 5J). Nano-(AZD + PD) treatment also led to a substantial increase in the percentage of GzmB⁺ and IFNγ⁺ CD8^+^ T cells, indicating enhanced cytotoxic function compared to the free drug combination or vehicle (Fig. 5K and Fig. S3). To rule out any antitumor effects from the nanocarrier itself, we treated MOC2 tumor-bearing C57BL/6 mice with or without blank LNPs. This analysis demonstrated that blank LNPs neither affected tumor growth nor T cell-mediated antitumor immunity during the treatment period (Fig. S6), confirming that the observed therapeutic benefits are due to the active agents rather than the delivery vehicle.

#### Nano-(AZD + PD) exhibits superior antitumor efficacy in HNSCC PDOs

**Fig. 6:**
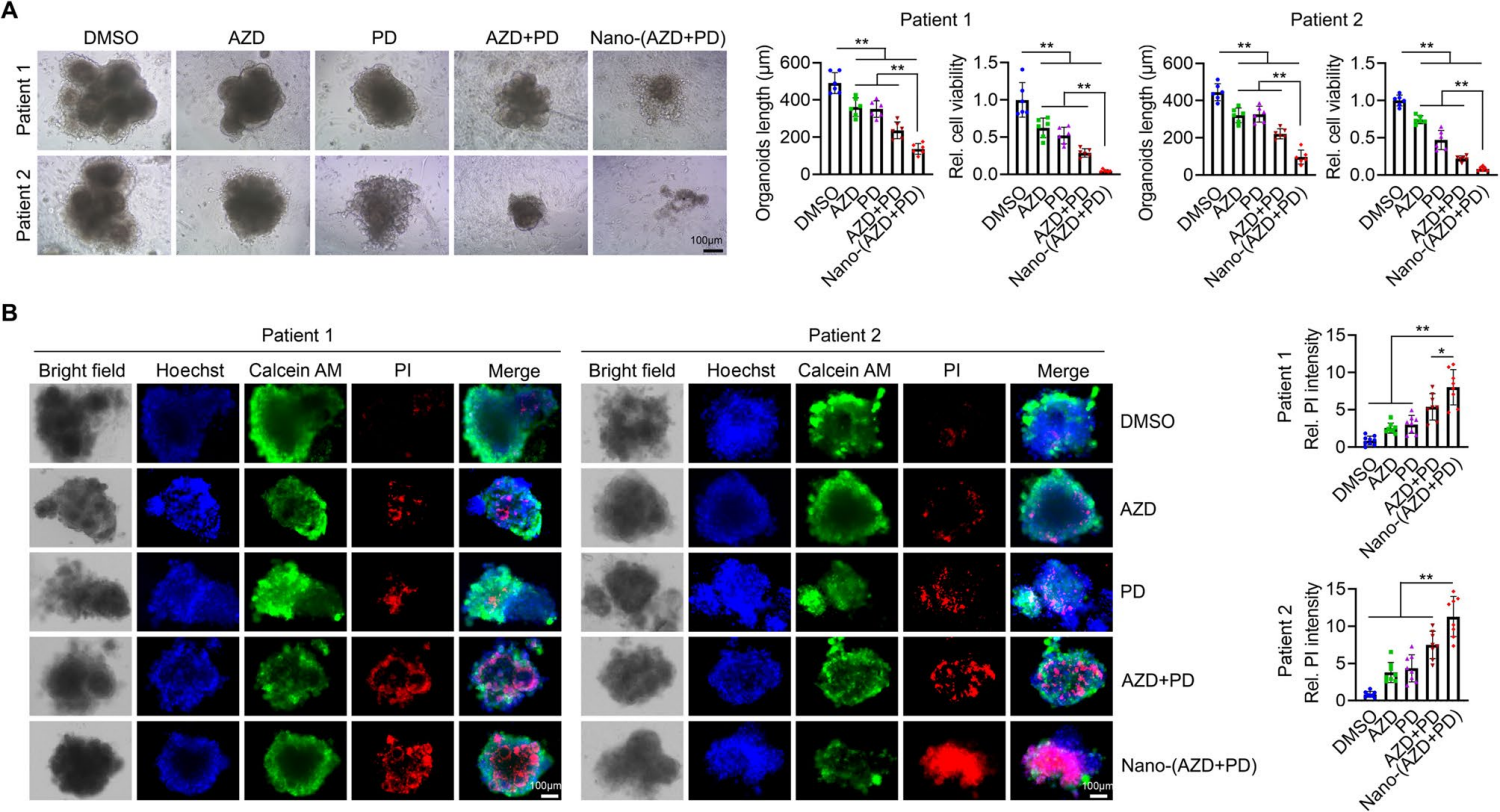
Nano-(AZD + PD) exhibits superior antitumor efficacy in HNSCC PDOs. **A** Effect of Nano-(AZD + PD) and other treatments on HNSCC PDO growth suppression. Organoids derived from two HNSCC patients were treated for 10 days. Representative images and quantitative data (*n* = 6 biological replicates/PDO) shown in the left and right panels, respectively. **B** HNSCC PDOs stained with Hoechst 33,342 (blue, nuclei), Calcein-AM (green, live cells), and PI (red, dead cells) after 5 days of the indicated treatments. Representative images and quantitative data (*n* = 8 biological replicates/PDO) shown in the left and right panels, respectively. Data are presented as mean values +/- SD. For statistical comparisons, *p* values were assessed by unpaired, two-tailed Student’s t test. *p < 0.05; **p < 0.01

PDOs have emerged as a more physiologically relevant in vitro model for studying human cancers, including HNSCC, as they more accurately recapitulate the complexity of the human tumor microenvironment (TME) [34, 35]. Our laboratory has established a panel of PDOs from HNSCC surgical specimens. Whole-genome sequencing (WGS) data revealed that two HPV (−) HNSCC PDOs harbor mutations in five of the most frequently mutated genes in this cancer subtype (Table S2). To evaluate the therapeutic efficacy of Nano-(AZD + PD), these two PDOs were treated with DMSO, AZD5438, PD0325901, the free drug combination, or Nano-(AZD + PD) (Fig. S7). Among all treatments tested, Nano-(AZD + PD) demonstrated the greatest efficacy in reducing PDO size and suppressing tumor cell viability (Fig. 6A). Additionally, PDOs treated with Nano-(AZD + PD) exhibited the highest propidium iodide (PI) staining intensity, further confirming its enhanced efficacy in inducing cell death (Fig. 6B). These findings provide strong evidence for the superior antitumor activity of Nano-(AZD + PD) in HNSCC and highlight its potential for improving therapeutic outcomes in patients with this cancer type.

## Discussion

Cancers characterized by elevated mutation rates display pronounced genomic instability, which contributes to intratumoral heterogeneity, activation of redundant signaling pathways, and tumor immune evasion [36–38]. These features abrogate the efficacy of monotherapies, as cancer cells can rapidly adapt and develop resistance through compensatory molecular mechanisms [39, 40]. Combination therapy has emerged as a well-established strategy to address these challenges by concurrently targeting multiple oncogenic pathways, thereby reducing the likelihood of resistance and enhancing therapeutic outcomes [41, 42]. Our data show that PD0325901 sensitivity is increased in HPV (−) HNSCC harboring *FAT1*, *CDKN2A*, or *NOTCH1* mutations, but decreased in tumors with other frequently mutated genes, including *TP53*, *TTN*, and *CSMD3* (Fig. 1B), suggesting that these mutations may confer PD0325901 resistance through alternative mechanisms. Interestingly, the addition of AZD5438 appears to restore or enhance drug efficacy in these resistant cases, supporting the notion that CDK inhibition may overcome PD0325901 resistance by targeting complementary cell cycle pathways. Our data further show that the combination of AZD5438 and PD0325901 elevates ROS levels, promoting Caspase 8 and GSDME cleavage and increasing HMGB1 secretion, thereby triggering pyroptosis and enhancing antitumor immunity. ROS elevation by AZD5438 alone may stem from cell cycle arrest–induced stress, which halts cell cycle progression and activates stress responses. When combined with MEK inhibition by PD0325901, which disrupts cell survival and proliferation signaling, this stress is amplified, causing metabolic imbalance and impaired antioxidant defenses that further increase ROS-associated pyroptosis. As highly mutated cancers often evade immune surveillance by downregulating antigen presentation, creating an immunosuppressive tumor microenvironment, and resisting T cell-mediated killing [43, 44], inducing pyroptosis through the combination of PD0325901 and AZD5438 in these cancers represents a promising strategy to simultaneously restrict tumor growth and enhance antitumor immunity.

However, despite its potential, combination therapy faces significant limitations, including divergent pharmacokinetic properties and half-lives of individual drugs, which can lead to suboptimal drug exposure and diminished synergistic effects [45, 46]. To overcome these limitations, nanoparticle-based drug delivery systems represent a promising solution for the co-delivery of multiple therapeutic agents [21, 47, 48]. Encapsulation within nanoparticles facilitates controlled and synchronized drug release, ensuring that multiple agents achieve therapeutically effective concentrations within the same cancer cell population. This approach not only overcomes drug resistance by concurrently targeting distinct pathways essential for tumor survival and progression but also optimizes synergistic interactions. To achieve precise co-delivery of AZD5438 and PD0325901 to the same individual cells in a 2:1 weight ratio, we employed nanotechnology to encapsulate both drugs within LNPs, resulting in the formulation of Nano-(AZD + PD). CryoEM analysis confirmed that Nano-(AZD + PD) adopts a vesicular structure, a morphology commonly observed in FDA-approved LNP-based drug formulations [49]. Lipid vesicles, or liposomes, are highly versatile carriers capable of encapsulating a broad spectrum of small-molecule drugs with diverse physicochemical properties. Hydrophobic drugs, such as AZD5438, can be integrated into the lipid bilayer via hydrophobic interactions, while hydrophilic drugs, like PD0325901, can be loaded into the aqueous core. Our studies demonstrated a rapid release profile for both AZD5438 and PD0325901 from Nano-(AZD + PD). Notably, AZD5438 displayed a faster release rate than PD0325901, likely due to weaker supramolecular interactions with the lipid components. However, it is critical to acknowledge that in vitro release conditions may not fully recapitulate the complex dynamics of the TME. Further optimization of the lipid composition and structure is necessary to achieve an optimal release profile that ensures synchronized delivery and maximizes therapeutic efficacy.

Despite the limitations of systemic delivery, intratumoral administration of the LNP-formulated combination of AZD5438 and PD0325901 achieved markedly greater tumor inhibition than the free drug combination. This enhanced efficacy likely results from co-encapsulation of both agents within the same LNPs, ensuring their simultaneous delivery to individual tumor cells and promoting synergistic interactions. In contrast, free drug administration allows each compound to diffuse and be taken up independently, reducing the likelihood of concurrent action within the same target cells. Intratumoral injection delivers therapeutics directly into the tumor, overcoming the limitations of poor systemic delivery and off-target toxicity by providing localized and concentrated drug exposure [29, 50]. This strategy is particularly advantageous in HNSCC, where tumors are often readily accessible, allowing precise administration. The LNP-based co-delivery system further enhances this approach by simultaneously delivering multiple agents in a controlled and efficient manner, improving drug stability, cellular uptake, and retention within the TME.

We observed a remarkable difference in the treatment efficacy of AZD5438 and PD0325901 combination between *Tcra* KO and wild-type C57BL/6 mice. Since CD8⁺ T cells are critical mediators of antitumor immunity and likely underlie much of this disparity, we focused our analysis on clarifying their specific contribution to the effects of dual CDK and MEK inhibition. We acknowledge, however, that other immune populations, including CD4⁺ T cells and dendritic cells, were not assessed. Considering the complexity of the tumor immune microenvironment, future studies will be designed to investigate these additional immune subsets, enabling a more comprehensive understanding of how diverse immune cells collectively modulate treatment efficacy and tumor control.

As a transformative tool in preclinical drug testing and personalized medicine, PDOs offer key advantages over conventional models, such as immortalized cell lines and animal models [51, 52]. PDOs are derived directly from patient tumor tissues, preserving the genetic, molecular, and phenotypic heterogeneity of the original tumors. Importantly, PDOs enable the prioritization of promising drug candidates for clinical trials, reducing the risk of late-stage failures and accelerating the drug development pipeline. In this context, the combination of AZD5438 and PD0325901 has demonstrated promising therapeutic efficacy in PDOs derived from surgical specimens of HNSCC patients, highlighting its potential for future clinical translation. Despite their advantages, PDOs face several challenges, including the need to maintain the full complexity of the TME, standardize culture conditions, and ensure scalability for widespread clinical application [53]. To address these limitations, one of our follow-up studies will focus on incorporating stromal and immune cells into PDO cultures to better recapitulate the TME. This approach will allow us to assess the impact of the AZD5438 and PD0325901 combination on immune cell infiltration, activation, and overall immunomodulatory effects within the TME. Another limitation of our study is the small number of PDOs analyzed in the present study, which may restrict the generalizability of our findings. Our proof-of-concept experiments were conducted in PDOs harboring mutations in *TP53*, *TTN*, *FAT1*, *CDKN2A* and *CSMD3*. Since HNSCC can present with diverse mutation combinations and tumors harboring all these five mutations are uncommon, future studies evaluating the efficacy of the AZD5438 and PD0325901 combination across PDOs with varying mutation profiles will be essential to strengthen the translational relevance of this treatment.

## Conclusions

In conclusion, this study demonstrates that the combination of AZD5438 and PD0325901 represents a promising therapeutic strategy for HNSCC, particularly in tumors with high mutational burden. The synergy between these agents not only enhances direct antitumor activity but also amplifies pyroptosis-associated antitumor immunity. These findings provide a strong rationale for the design of clinical trials aimed at evaluating the safety, tolerability, and efficacy of this combination in patients with high-mutation HNSCC.

## Supplementary Information

Supplementary Material 1.

## Data Availability

The datasets used and/or analyzed during the current study are available from the corresponding author on reasonable request.

## Abbreviations

CDK: Cyclin-dependent kinase
CF: Correlation factor
DMG-PEG2000: 1,2-dimyristoyl-rac-glycero-3-methoxypolyethylene glycol-2000
DOPC: 1,2-dioleoyl-sn-glycero-3-phosphocholine
ERK: Extracellular signal-regulated kinase
GSDMD: Gasdermin D
GSDME: Gasdermin E
HNSCC: Head and neck squamous cell carcinoma
HMGA1: High mobility group at-hook 1
HMGB1: High mobility group box 1
HPV (−): Human papillomavirus negative
HPV (+): Human papillomavirus positive
LC-MS: Liquid chromatography-mass spectrometry
LDH: Lactate dehydrogenase
LNPs: Lipid nanoparticles
IFNγ: Interferon-gamma
IHC: Immunohistochemistry
MAPK: Mitogen-activated protein kinase
PDOs: Patient-derived organoids
Rb: Retinoblastoma protein
TME: Tumor microenvironment
