# Supplementary material for: Dual CDK and MEK Inhibition potentiates CD8+ T cell-mediated antitumor immunity by inducing pyroptotic cell death in high-mutational head and neck cancer
Source: J Exp Clin Cancer Res. 2025 Nov 6;44:300. doi: 10.1186/s13046-025-03557-7 (PMC12590917; doi:10.1186/s13046-025-03557-7)
Supplement: Supplementary file 1 — Supplementary Material 1. [file 13046_2025_3557_MOESM1_ESM.docx]

**Supplemental Information for**

**Dual CDK and MEK inhibition potentiates CD8^+^ T cell-mediated antitumor immunity by inducing pyroptotic cell death in high-mutational head and neck cancer**

Fanghui Chen^1†^, Fang Yang^1†^, David O. Popoola^2^, Jianqiang Yang^1^, Chris Tang^1^, Alexis Payne^1^, Lynn Zhang^1^, Nicole C. Schmitt^3,4^, Jin Xie^5^, Nabil F. Saba^1,4^, Yamin Li^2*^, Yong Teng^1,4,6*^

**Correspondence:** Yong Teng, [yong.teng@emory.edu](mailto:yong.teng@emory.edu)

Yamin Li, [liyam@upstate.edu](mailto:liyam@upstate.edu)

This file includes:

Supplementary Tables (Table S1 and Table S2)

Supplementary Figures (Figure S1-Figure S7)

**Table S1. IC_50_, HillSlope and logIC_50_ of the two drugs in four HNSCC cell lines**

| **HN12** |  |  |  | **SCC15** |  |  |
| --- | --- | --- | --- | --- | --- | --- |
|  | AZD5438 | PD0325901 |  |  | AZD5438 | PD0325901 |
| IC_50_ | 3.852 | 2.07 |  | IC_50_ | 4.787 | 3.229 |
| HillSlope | -0.8103 | -0.9104 |  | HillSlope | -0.9357 | -0.8158 |
| logIC_50_ | 0.5857 | 0.3159 |  | logIC_50_ | 0.68 | 0.5091 |
|  |  |  |  |  |  |  |
| **Cal27** |  |  |  | **SCC1** |  |  |
|  | AZD5438 | PD0325901 |  |  | AZD5438 | PD0325901 |
| IC_50_ | 2.988 | 1.729 |  | IC_50_ | 5.881 | 4.712 |
| HillSlope | -1.051 | -0.9125 |  | HillSlope | -1.026 | -0.76 |
| logIC_50_ | 0.4754 | 0.2378 |  | logIC_50_ | 0.7695 | 0.6732 |

**Table S2. Gene mutations of PDO_HNSCC#1 and PDO_HNSCC#2**

| **PDO_HNSCC#1** | **Mutations** | ***TP53*** | ***TTN*** | ***FAT1*** | ***CDKN2A*** | ***CSMD3*** |
| --- | --- | --- | --- | --- | --- | --- |
|  | **CNVs** | - | - | - | - | + |
|  | **Indels** | + | + | + | + | + |
|  | **SNPs** | - | + | - | - | - |
|  | **SVs** | - | - | - | - | + |
| **PDO_HNSCC#2** | **CNVs** | - | - | - | - | + |
|  | **Indels** | + | + | + | + | + |
|  | **SNPs** | - | + | - | - | - |
|  | **SVs** | - | - | - | - | + |

Note:

“-”: no mutations; “+”: mutations; CNVs: copy number variants; InDels: small insertions and deletions; SNPs: single nucleotide polymorphisms; and SVs: structural variants.

**Figure S1. Analysis of synergistic effects of AZD5438 and PD0325901 in various HNSCC cell lines.** (**A**) Combination treatment effects of AZD5438 with PD0325901 at the indicated doses (72 hours) on the viabilities of Cal27, HN12, SCC1, and SCC15 cells. Each block represents the mean cell viability inhibition with standard deviation (SD) (n = 3). (**B**) A three-dimensional (3D) plot showing the Loewe synergy score for pairwise dose combinations in Cal27, HN12, SCC1, and SCC15 cells. z axis, synergy score; x/y axis, drug combination with different doses.

**
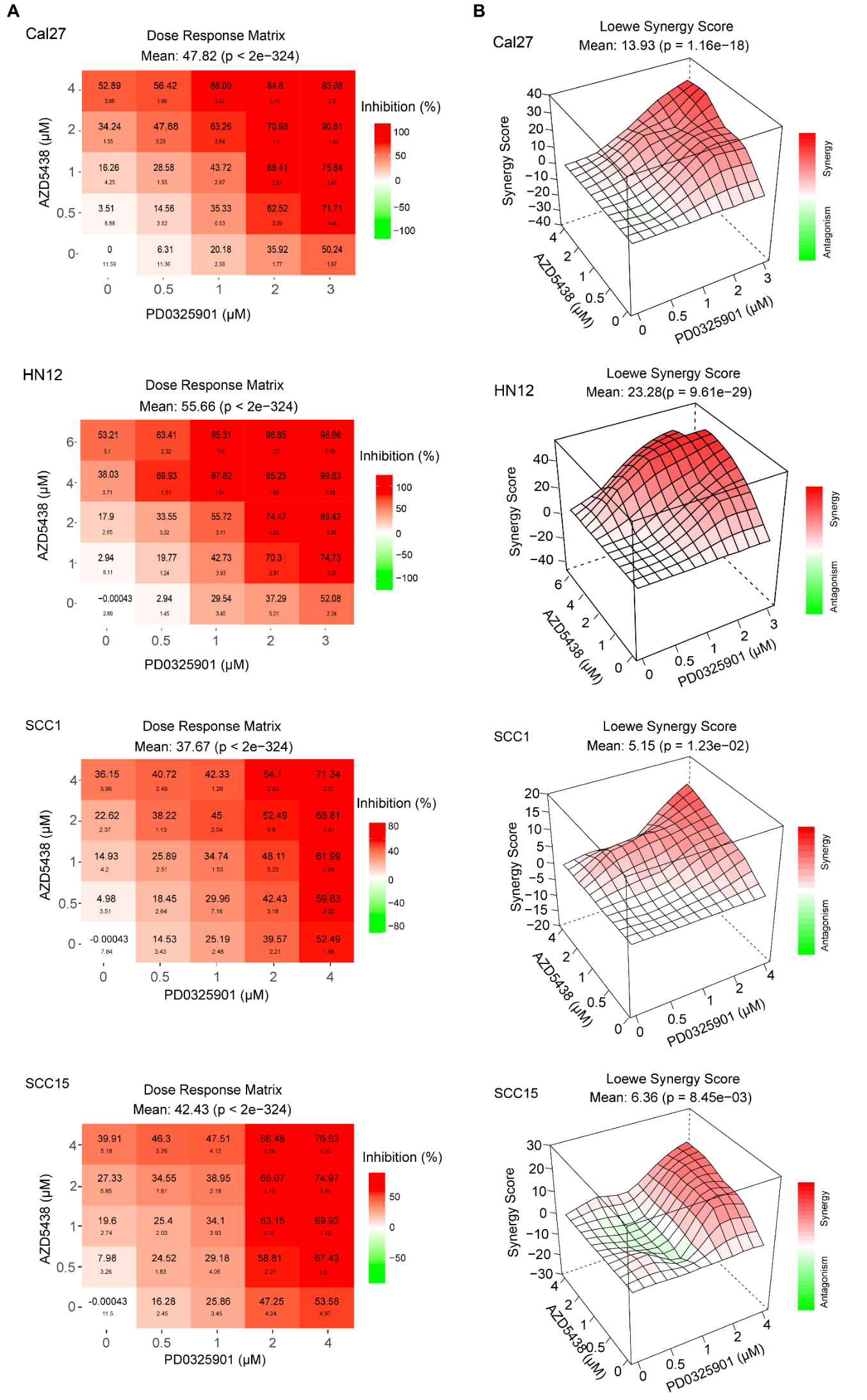
**

**Figure S2. Quantification of Western blot data.** (**A**) Quantitative analysis of Western blot results (n = 3 biological replicates) corresponding to the representative images shown in Fig. 2D. (**B**) Quantitative analysis of Western blot results (n = 3 biological replicates) corresponding to the representative images shown in Fig. 3F. Data are presented as mean values +/- SD. For statistical comparisons, *p* values were assessed by unpaired, two-tailed Student’s t test. **p*<0.05; ***p*<0.01.


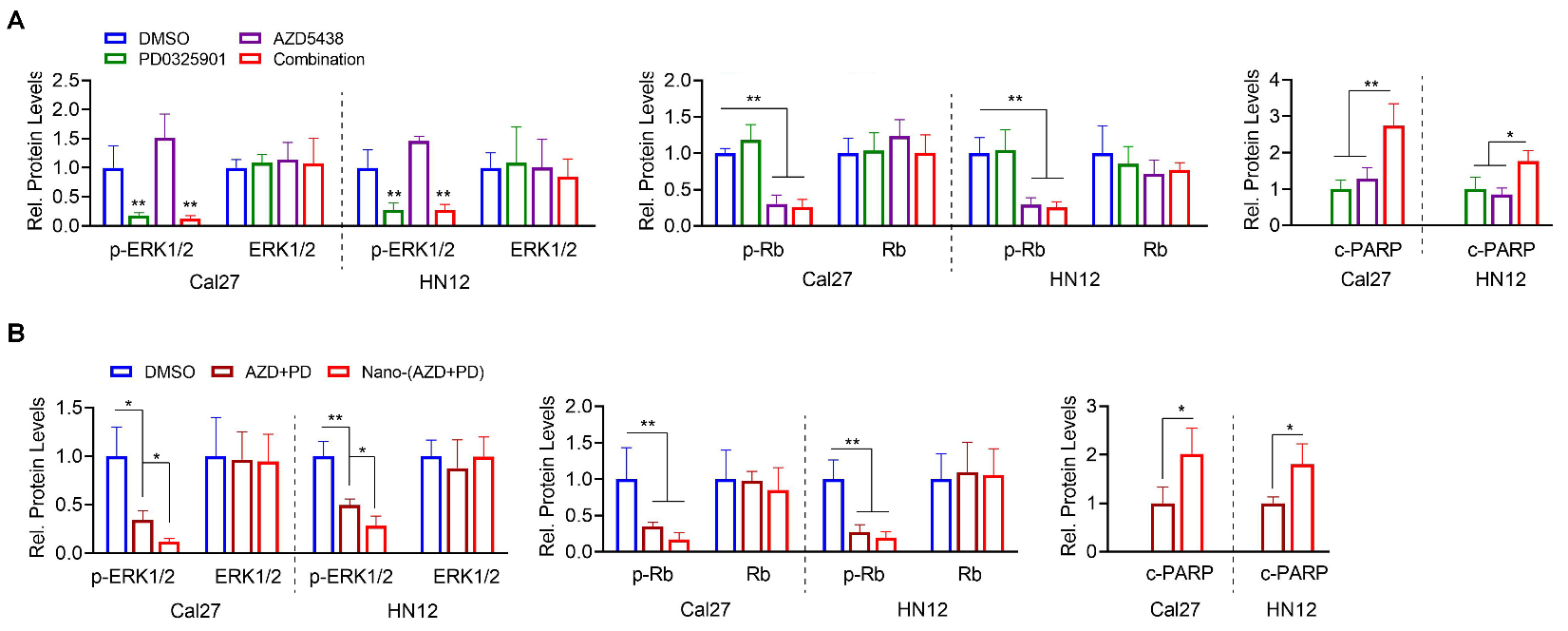


**Figure S3. The flow gating strategies for apoptosis (Fig. 2E), ROS (Fig. 4F and 5B), and CD8^+^ T cells (Fig. 5I and 5K).** (**A**) For assessing apoptotic rate, cells were gated to exclude debris by employing forward scatter area (FSC-A) in conjunction with side scatter area (SSC-A), following by the discrimination of doublets using forward scatter height versus forward scatter area (FSC-H vs. FSC-A). Viable single cells were subjected to apoptosis analysis through PE-Annexin V staining, and mean fluorescence intensity (MFI) of PE-Annexin V was quantified. (**B**) For measuring intracellular ROS levels, cells were first gated to exclude debris based on forward scatter area (FSC-A) versus side scatter area (SSC-A). Doublets were excluded by gating on forward scatter height versus forward scatter area (FSC-H vs FSC-A). ROS levels were assessed in the single-cell population following DCF staining, and MFI was quantified. (**C**) For assessing CD8^+^ cells, the gating strategy began with the exclusion of debris based on forward scatter area (FSC-A) versus side scatter area (SSC-A). Doublets were discriminated using FSC height versus FSC area (FSC-H vs FSC-A). Live cells were identified by excluding dead cells using a Live/Dead viability dye. CD3⁺ T cells were then gated from the live cell population, followed by analysis of CD8⁺ T cell subsets for further analysis.


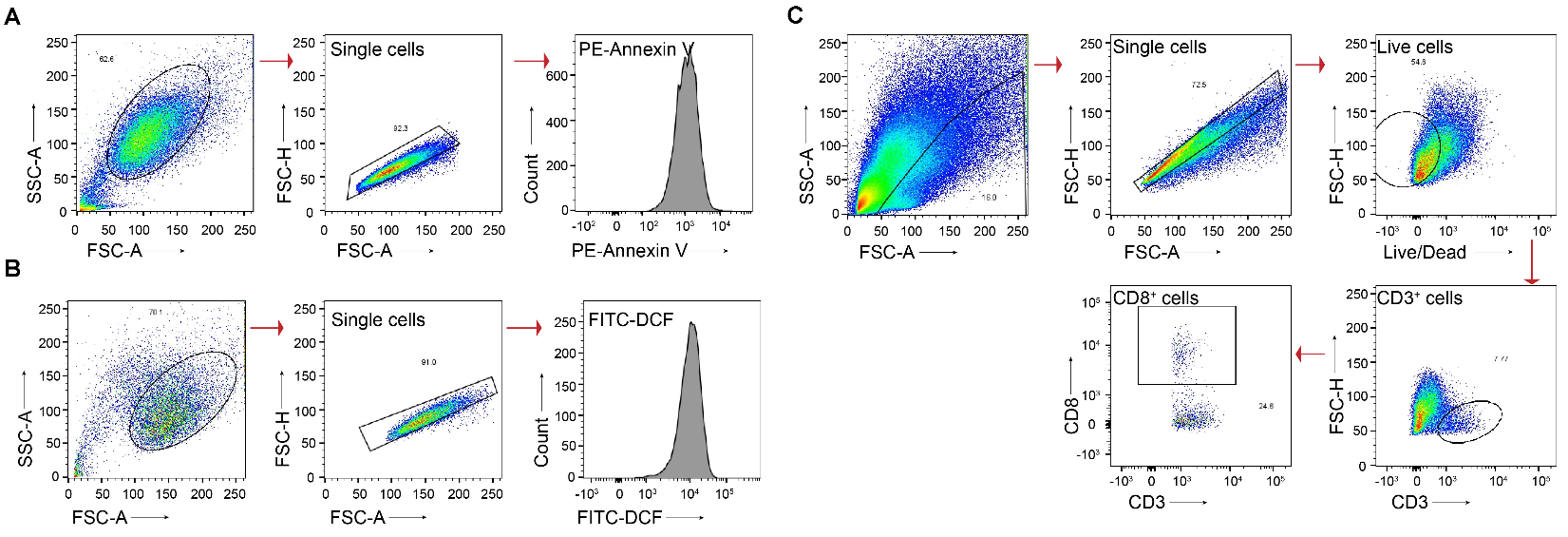


**Figure S4. The combination of AZD5438 and PD0325901 does not result in cytotoxicity in the major organs.** Histopathological analysis of the livers and lungs extracted from HN12 tumor-bearing NSG mice treated with vehicle, Nano-(AZD+PD), or the free drug combination.

**
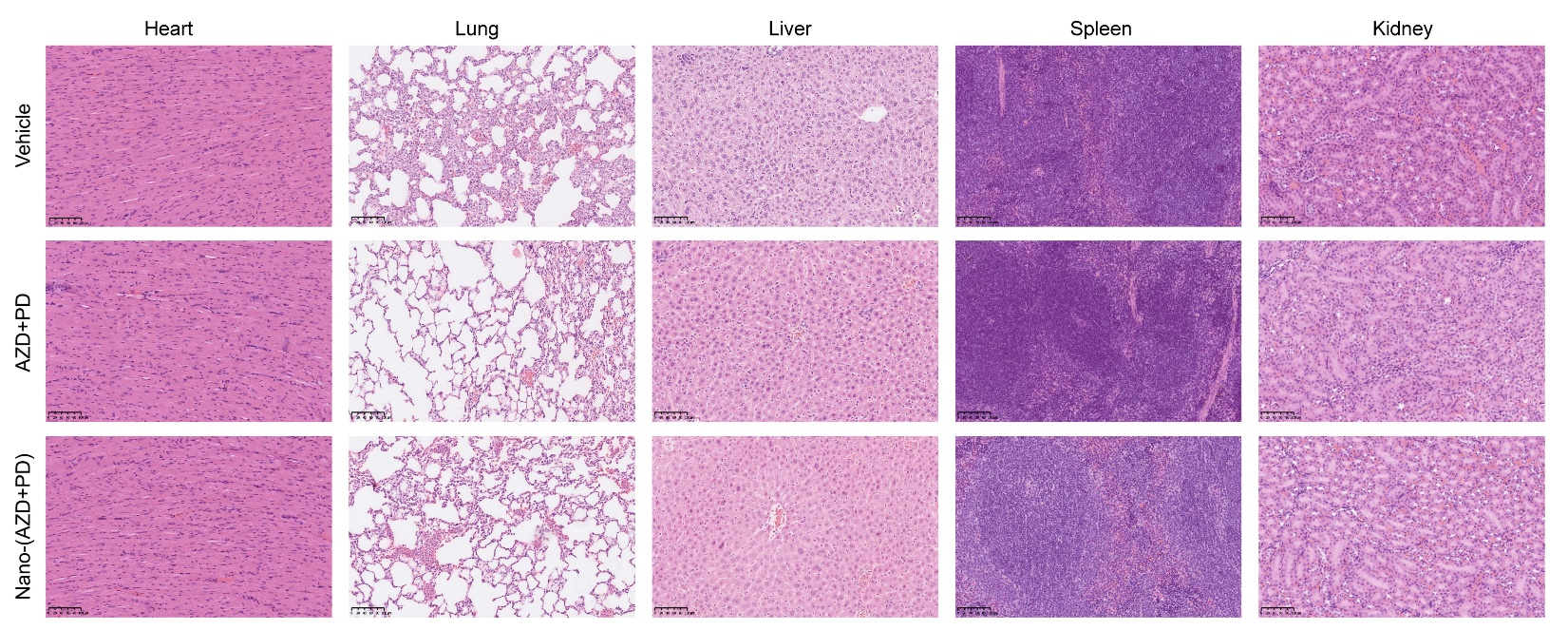
**

**Figure S5. The combination of AZD5438 and PD0325901 induces pyroptotic cell morphology in HNSCC cells.** (**A**) Morphological changes in Cal27 and HN12 cells treated with DMSO, AZD5438 and PD0325901, alone in combination. (**B**) Morphological changes in Cal27 and HN12 cells treated with DMSO, the free drug combination, or Nano-(AZD+PD). In (A) and (B), representative microscopic images and quantitative data (n = 20 views) are shown in the left and right panels, respectively. Data are presented as mean values +/- SD. For statistical comparisons, *p* values were assessed by unpaired, two-tailed Student’s t test. ****p*<0.001.

**
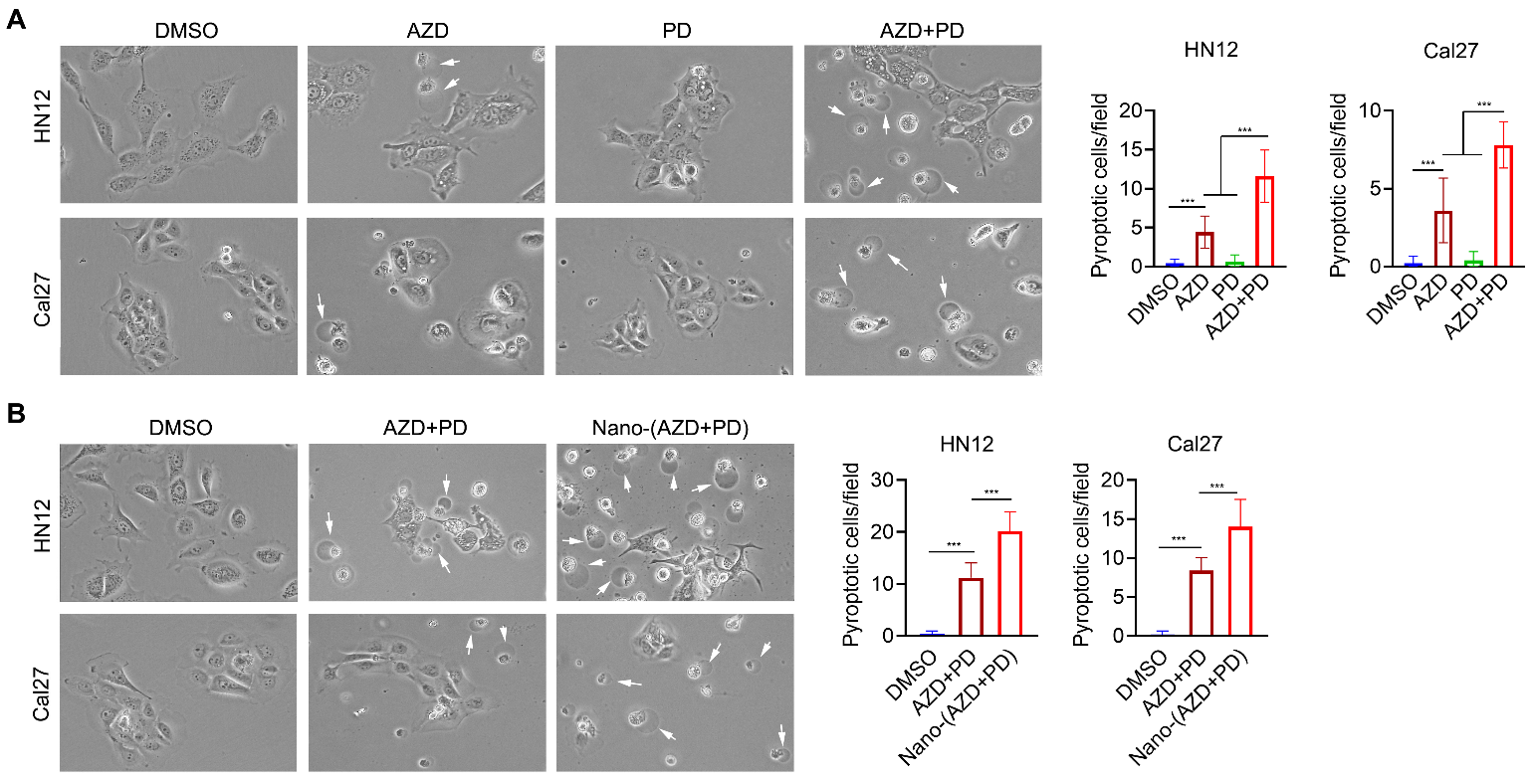
**

**Figure S6. Blank nanocarrier does not affect tumor development and CD8^+^ T cell-mediated immunity.** (**A, B**) Tumor growth curve and weight (A) and body weight (B) in mice receiving vehicle or blank LNPs treatment (n = 5 mice/group). (**C**) Percent of CD8^+^ T cells and its cytotoxic (GzmB^+^) subset in MOC2 tumor receiving vehicle or blank LNPs treatment. An orthotopic MOC2 tumor model was established in the buccal mucosa of C57BL/6 mice, followed by intratumoral administration of either vehicle or blank LNPs every other day for a total of seven doses. Tumors were excised on Day 28 after cell inoculation for flow cytometry analysis.


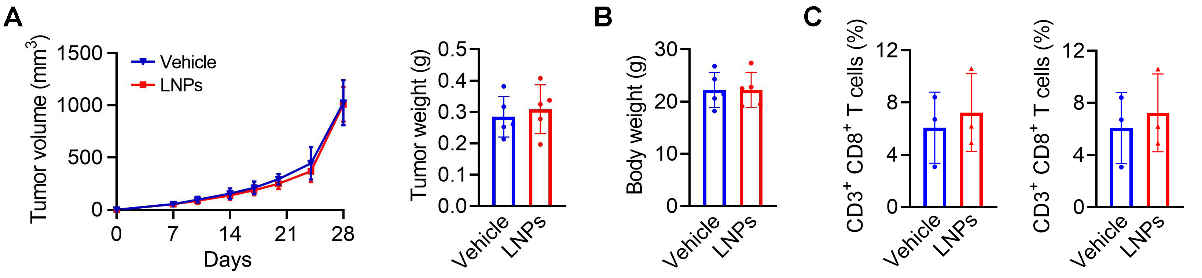


**Figure S7. Schematic diagram depicting the process of developing HNSCC patient-derived organoids for drug treatment. Figure created using Biorender (**[**https://biorender.com**](https://biorender.com)**).**

**
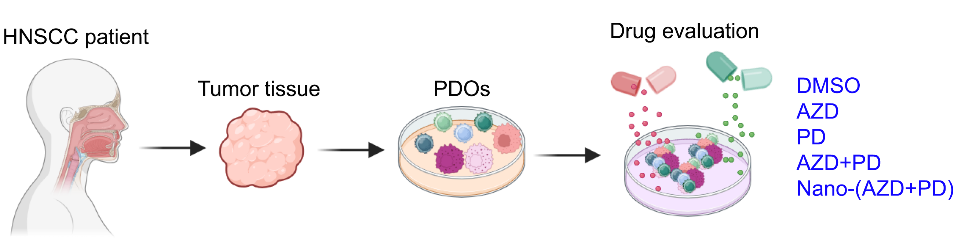
**
